# Supplementary material for: Rapid turnover of life-cycle-related genes in the brown algae
Source: Genome Biol. 2019 Feb 14;20:35. doi: 10.1186/s13059-019-1630-6 (PMC6374913; doi:10.1186/s13059-019-1630-6)
Supplement: Supplementary file 3 — Protein alignments, corresponding Gblocks html file and CDS sequences for the lineage conserved gametophyte-biased genes. (ZIP 2109 kb) [file 13059_2019_1630_MOESM3_ESM.zip › Con_GA_alignments/Ec-26_004110.aa_ali.fasta-gb.htm]

Ec-26\_004110.aa.fasta.trans.aa\_ali.fasta


## Gblocks 0.91b Results

Processed file: **Ec-26\_004110.aa.fasta.trans.aa\_ali.fasta**  
Number of sequences: **2**  
Alignment assumed to be: **Protein**  
New number of positions: **324** (selected positions are underlined in blue)

```
                         10        20        30        40        50        60
                 =========+=========+=========+=========+=========+=========+
Ec-26_004110     MGIVVRRAEPGDQRGLTTLVGKAGGPSMFRKLFGSYNFGQMIETSYLSVSALSTLDPNPA
MSTRG.40490.1    MGIIVRRAEPGDQRGLTTLVGKAGGPSMFRKLFGSYNFGQMIETSYLSVSALSAVSADP-
                 ########################################################### 


                         70        80        90       100       110       120
                 =========+=========+=========+=========+=========+=========+
Ec-26_004110     GGGDVHLPGNR--QQQHPEFLVGFAILSDSPRAGISEDWLEWFSETHAPADAEVTMTNTL
MSTRG.40490.1    GGGDVRSAGDRSQQKQPPEILVGFAILSDSPRAGVSEDWLEWFSETHAPADAEVTMTNTL
                 ###########  ###############################################


                        130       140       150       160       170       180
                 =========+=========+=========+=========+=========+=========+
Ec-26_004110     WMDFAVAVSAARASGVVSGVAVGREEEGDNNAAARRGEEAEEEEAAGILEDIVRTVFNTL
MSTRG.40490.1    WVDFAIAISPARAA-ASGGLAAGREEGGDDATAQ----DEEEEAAAGVIEDIVRTVFNTL
                 #############     ###############       ####################


                        190       200       210       220       230       240
                 =========+=========+=========+=========+=========+=========+
Ec-26_004110     PEIDYLVISLPGDTSTGGKGS--KKGAGTASIPTPAYLLRAFEVLSRHADASPNNLDEAY
MSTRG.40490.1    PEVDYLVMSLPGNPGAGGGGSGKKSAAGPANVPTPAYLLRAFEVLGRHADASPNNLDEAY
                 #####################  #####################################


                        250       260       270       280       290       300
                 =========+=========+=========+=========+=========+=========+
Ec-26_004110     GTPRLLLCDRMAFLPTLAIRPACVEDHDDLMPVFAAQSDLLSATYGEFFLADMIELQDNE
MSTRG.40490.1    GKPLLLLCDRMAFLPTLAVRPACVEDHDDLMPVFAAQSDLLSATYGEFFLADMIELQDSE
                 ############################################################


                        310       320       330       340       350       360
                 =========+=========+=========+=========+=========+=========+
Ec-26_004110     NKALAALVQGRTCGLLATSSDLELGLLQSCFQLDDYEHLVKASEAPRVLILGPPGAGKTT
MSTRG.40490.1    NKALAALVQGRACGLLATSSDLELGLLQSCFQLDDYEHLVKV------------------
                 #########################################                   


                        370       380       390       400       410       420
                 =========+=========+=========+=========+=========+=========+
Ec-26_004110     LAVAMAAKYGSVLVSVDAEAKAAADAGSEQGERALKLLDAGEVLPSEMVLSLVATKLASR
MSTRG.40490.1    ------------------------------------------------------------
                                                                             


                        430       440       450
                 =========+=========+=========+======
Ec-26_004110     ECQKSGWVLEGVGTAATGVDELEEAIVMATEVLVIA
MSTRG.40490.1    ------------------------------------
```

```
Parameters used
Minimum Number Of Sequences For A Conserved Position: 2
Minimum Number Of Sequences For A Flanking Position: 2
Maximum Number Of Contiguous Nonconserved Positions: 8
Minimum Length Of A Block: 10
Allowed Gap Positions: None
Use Similarity Matrices: Yes
```

```
Flank positions of the 6 selected block(s)
Flanks: [1  59]  [61  71]  [74  133]  [139  153]  [161  201]  [204  341]  

New number of positions in Ec-26_004110.aa.fasta.trans.aa_ali.fasta-gb:  324  (71% of the original 456 positions)
```
